# Molecular Changes Induced in Melanoma by Cell Culturing in 3D Alginate Hydrogels

**DOI:** 10.3390/cancers13164111

**Published:** 2021-08-15

**Authors:** Melanie Kappelmann-Fenzl, Sonja K. Schmidt, Stefan Fischer, Rafael Schmid, Lisa Lämmerhirt, Lena Fischer, Stefan Schrüfer, Ingo Thievessen, Dirk W. Schubert, Alexander Matthies, Rainer Detsch, Aldo R. Boccaccini, Andreas Arkudas, Annika Kengelbach-Weigand, Anja K. Bosserhoff

**Affiliations:** 1 Faculty of Computer Science, Deggendorf Institute of Technology, Dieter-Görlitz-Platz 1, 94469 Deggendorf, Germany; melanie.kappelmann-fenzl@th-deg.de (M.K.-F.); stefan.fischer@th-deg.de (S.F.); 2 Institute of Biochemistry, Friedrich-Alexander University Erlangen-Nürnberg (FAU), Fahrstraße 17, 91054 Erlangen, Germany; sonja.s.schmidt@fau.de (S.K.S.); lisa.laemmerhirt@fau.de (L.L.); alexander.matthies@fau.de (A.M.); 3 Department of Plastic and Hand Surgery, University Hospital Erlangen, Friedrich-Alexander University Erlangen-Nürnberg (FAU), Krankenhausstraße 12, 91054 Erlangen, Germany; rafael.schmid@uk-erlangen.de (R.S.); Andreas.Arkudas@uk-erlangen.de (A.A.); Annika.Kengelbach-Weigand@uk-erlangen.de (A.K.-W.); 4 Institute of Physics, Friedrich-Alexander University Erlangen-Nürnberg (FAU), Henkestraße 91, 91054 Erlangen, Germany; lena.lf.fischer@fau.de (L.F.); ingo.thievessen@fau.de (I.T.); 5 Institute of Polymer Materials, Friedrich-Alexander University Erlangen-Nürnberg (FAU), Martensstraße 7, 91058 Erlangen, Germany; stefan.schruefer@fau.de (S.S.); dirk.schubert@fau.de (D.W.S.); 6 Institute of Biomaterials, Friedrich-Alexander University Erlangen-Nürnberg (FAU), Cauerstraße 6, 91058 Erlangen, Germany; rainer.detsch@ww.uni-erlangen.de (R.D.); Aldo.Boccaccini@fau.de (A.R.B.)

**Keywords:** melanoma, biomaterials, 3D cultivation, whole transcriptome, EGR1

## Abstract

**Simple Summary:**

The research field of 3D cell cultivation in hydrogels is continuously growing. To be able to analyze the reaction of melanoma cells to 3D cultivation in alginate hydrogel on a molecular level, whole transcriptome sequencing was performed. Intriguingly, we could not only unravel differences between the gene regulation in 2D and 3D cultures but could also correlate the culture switch to the physiological process of tumor plasticity based on the observed patterns. Thereby, the role of EGR1 in controlling tumor plasticity and progression in melanoma was revealed. We conclude that the combination of cell culture models using biomaterials and whole transcriptome analysis leads to a deeper molecular understanding of cancer cells, herewith defining new therapeutic targets.

**Abstract:**

Alginate hydrogels have been used as a biomaterial for 3D culturing for several years. Here, gene expression patterns in melanoma cells cultivated in 3D alginate are compared to 2D cultures. It is well-known that 2D cell culture is not resembling the complex in vivo situation well. However, the use of very intricate 3D models does not allow performing high-throughput screening and analysis is highly complex. 3D cell culture strategies in hydrogels will better mimic the in vivo situation while they maintain feasibility for large-scale analysis. As alginate is an easy-to-use material and due to its favorable properties, it is commonly applied as a bioink component in the growing field of cell encapsulation and biofabrication. Yet, only a little information about the transcriptome in 3D cultures in hydrogels like alginate is available. In this study, changes in the transcriptome based on RNA-Seq data by cultivating melanoma cells in 3D alginate are analyzed and reveal marked changes compared to cells cultured on usual 2D tissue culture plastic. Deregulated genes represent valuable cues to signaling pathways and molecules affected by the culture method. Using this as a model system for tumor cell plasticity and heterogeneity, EGR1 is determined to play an important role in melanoma progression.

## 1. Introduction

Cell culture models have contributed decisively to our contemporary knowledge of cell biology in healthy and diseased organisms. Besides the longstanding two-dimensional (2D) culture, there is a continuous trend towards culturing cells in three dimensions (3D). By cultivating cells encapsulated in hydrogels, the physiological conditions including proliferation rates, metabolism, cell morphology, and extracellular matrix (ECM) production can be represented more accurately than on flat surfaces [1,2,3,4]. This is not only relevant for biomedical applications like tissue engineering, but also the detailed investigation of cellular effects in cancer research and drug development. Here, one major advantage of 3D culture models compared to in vivo animal models is their rather low complexity, making them highly definable and allowing researchers to selectively modulate the system and analyze molecular aspects in an isolated manner. After analysis, animal models and patient data are the means of choice to substantiate and confirm the in vitro results (reviewed in [5]).

Alginate is widely used as a polysaccharide hydrogel for the 3D culture of cancer cells. It has favorable properties like being non-cytotoxic, non-immunogenic, and biodegradable and, additionally, alginate provides the ability to be chemically modified via its native free hydroxyl or carboxyl functional groups [6,7]. As reviewed by Neves et al. in detail [8], various modifications like e.g., oxidation, thiolation, and esterification can be performed. Thereby, e.g., mechanical properties and the degradation rate of the material can be tailored directly or additional bioactive molecules, like cell adhesive peptides, can be linked to the otherwise non-adhesive material. Hence, alginate and different derivatives are applied in various contexts including studies on cellular behavior, metabolism, and compound screening [9,10,11].

Despite the broad range of application areas and the tremendous efforts put into modifying and improving the alginate matrix for 3D cell culture applications, the basic molecular differences between the common 2D system and 3D alginate culture are yet to be defined and understood. This emphasizes the need for molecular analyses, helping all researchers in this interdisciplinary field. Most studies focus on the exclusive analysis of a selection of genes associated with observed phenotypic changes like cellular adhesion and ECM. Cavo et al. [12] embedded the breast cancer cell line MCF-7 in alginate, and clearly demonstrated the altered, more physiological morphology of cells or multicellular structures in comparison to those observed by 2D cultivation on a petri dish or alginate by optical analysis. In a previous study, we were able to demonstrate a similar behavior of melanoma cell lines Mel Im and MV3 in 3D alginate [13]. We suggest that unraveling the differences between the whole transcriptomic expression patterns of these two conditions by RNA sequencing is crucial for the interpretation of the observed cellular behavior. In-depth analysis of RNA-seq data thereby allows the identification of gene expression, molecular mechanisms, and biological processes in an unbiased manner [14,15].

In our study, we use malignant melanoma as a model system. Melanoma is characterized by early and aggressive metastasis already from small primary tumors. Once metastasized to the lymph nodes, cancer poses a severe threat to the patient, as further spreading to distant organs and tumor recurrence become even more likely [16,17]. To be used for the analysis of the physiological mechanisms contributing to the disease pattern and to facilitate broader target and drug screening, 3D cell culture models have to be defined and deciphered in detail.

This study compares the gene expression patterns of melanoma cells cultivated in a 2D culture dish to cultivation in 3D alginate hydrogel, by bioinformatic analysis of RNA sequencing data, and points out how the specific expression level of cells cultured in 0.6% (*w/v*) alginate may contribute to the more physiological behavior in this setting. 

## 2. Materials and Methods

### 2.1. Cell Lines and Cell Culture Conditions

All cell lines used have been described previously [18]. The human melanoma cell lines Mel Wei, A375 (derived from a primary cutaneous tumor), Mel Im, Mel Ju, and SkMel28 (derived from malignant melanoma metastases) were cultivated in Dulbecco’s Modified Eagle’s Medium (DMEM) low glucose, supplemented with 10% fetal calf serum (FCS), penicillin (400 U/mL) and streptomycin (50 µg/mL) (all from Sigma Aldrich, St. Louis, MO, USA). The primary melanoma cell line Mel Juso was cultivated in Roswell Park Memorial Institute-culture media (RPMI) (Sigma Aldrich) with the same supplements. The cell lines were incubated at 37 °C in a humidified atmosphere containing 8% CO_2_.

For the cultivation of primary melanoma cell line WM3211, the culture medium consisted of MCDB153 (Sigma Aldrich) with 20% Leibowitz’s L-15 (PAA Laboratories, Pasching, Austria), 2% FCS, 1.68 mM CaCl_2_ (Sigma Aldrich), 5 µg/mL insulin (Sigma Aldrich), penicillin (400 U/mL) and streptomycin (50 µg/mL) (Sigma Aldrich). Normal human epithelial melanocytes (NHEMs) were derived from neonatal foreskin and cultivated in a melanocyte growth medium (both from PromoCell, Heidelberg, Germany). These two cell lines were incubated at 37 °C in a humidified atmosphere containing 5% CO_2_.

The FUCCI (fluorescence ubiquitination-based cell cycle indicator) system was stably integrated into Mel Im via lentiviral transduction of plasmid pBOB-EF1-FastFUCCI-Puro, as described elsewhere [19,20]. Expression of the fluorescence coupled cell cycle proteins Chromatin licensing and DNA replication factor 1 (Cdt1) (red) or geminin (green) indicate either G1 or S/G2/M state of the cells. During the transition from G1 to S phase, both proteins are present and merge producing a yellow fluorescence signal xxx. The resulting cell line Mel Im FUCCI was cultivated like the parental cell line Mel Im. To avoid the loss of the reporter, 4 µg/mL puromycin (Sigma Aldrich) was added every second week for selection.

For 3D alginate hydrogels, VIVAPHARM^®^ Alginate PH 176 (JRS PHARMA GmbH & Co. KG, Rosenberg, Germany) was dissolved in PBS without Ca^2+^ and Mg^2+^ (Sigma-Aldrich) to a final concentration of 0.6% (*w/v*). A positive displacement pipette was used to re-suspend Mel Im or Mel Im FUCCI cells (3 × 10^5^ mL^−1^) in the hydrogel and pipet droplets of 300 µL of the cell suspension into a 6-well plate. Droplets were chemically crosslinked using a 50 mM CaCl_2_ solution (Sigma-Aldrich). After 10 min of crosslinking, the gelled constructs were washed with and subsequently covered with the appropriate cell culture medium and incubated at 37 °C and 8% CO_2_ for up to 7 days. For simplicity, the fabricated hydrogels are referred to as 0.6% (or 3D) alginate in this paper.

### 2.2. Fluorescence Microscopy

Microscopy of Mel Im FUCCI in 2D and 3D alginate was performed on days 1, 2, 4, and 7 after seeding using an Olympus IX83 fluorescence microscope (Olympus, Tokyo, Japan). Overlay images (2D) or z-stacks of 200 µm depth (3D alginate) were taken using the Olympus CellSens Dimension software (v 2.2, Olympus, Tokyo, Japan, 2009) in 3 biological replicates for 3D alginate, and 2 biological replicates for 2D culture. For each condition, 3 technical replicates were performed.

### 2.3. Cell Cycle Quantification

The cell cycle state of Mel Im FUCCI cells in 2D culture or 3D alginate was quantified based on fluorescence images taken on day 1 as described above. Using the Cell Counter plugin in the software Fiji is just ImageJ (v 1.53c) [21], red (G1), green (S/G2/M) or yellow (G1/S) cells were quantified. Three technical replicates were quantified in 2 independent experiments for 2D culture and 3 independent experiments for 3D alginate.

### 2.4. Proliferation Analysis

The cell proliferation of Mel Im FUCCI cells in 3D alginate was quantified based on fluorescence image z-stacks taken on days 1, 2, and 4 from 3 alginate constructs fabricated in 3 independent experiments as described above. Each image z-stack was merged into one image displaying the maximum fluorescence intensity over z, and cells per cluster were counted using the software Fiji is just ImageJ (v 1.53c) [21]. Summarized counts were compared to 2D proliferation values based on a doubling time of approx. 16 h observed for Mel Im in our lab.

### 2.5. Dynamic Mechanical Analysis (DMA)

Analysis of the viscoelastic properties of 0.6% alginate were performed as described previously [13]. Briefly, 0.6% alginate platelets were prepared in 12-well plates by pipetting 1 mL of the alginate solution between filter papers soaked with 50 mM CaCl_2_ for crosslinking (10 min). Gels were transferred into cell culture medium and incubated at 37 °C and 8% CO_2_ overnight before they were measured using a DHR-3 rheometer (TA Instruments, New Castle, DE, USA) in oscillating compression mode at 37 °C. The used gap distance is adjusted by the applied preload and ranged between 1300 and 1500 μm. A preload of 0.2 N and the used deformation of 1% were determined by preliminary amplitude sweeps. To analyze the storage modulus E’ and loss modulus E’’ a frequency sweep, covering the range of 0.1 to 100 rad/s is performed and evaluated for each sample. Four samples are averaged to ensure statistical significance.

### 2.6. RNA Isolation for RNA-Seq

RNA was isolated using TRIzol as described previously [22]. Briefly, 2 days after seeding Mel Im cell pellets or whole cell-laden alginate constructs were lysed in TRIzol and centrifuged to remove insoluble material. After phase separation with chloroform, the RNA was precipitated from the aqueous phase using 100% isopropanol. The resulting RNA pellet was washed with 75% ethanol, air-dried, and resuspended in elution buffer (Qiagen, Hilden, Germany).

### 2.7. RNA-Seq Library Preparation and Mapping

RNA-Seq libraries were prepared as described previously [15]. Library preparation was performed with three biological replicates. Sequencing was performed according to the paired-end RNA sequencing protocols from Illumina on a HiSeq4000 with a paired-end module (Illumina, Inc., San Diego, CA, USA). The samples were sequenced from each side of a fragment approximately 75 bp long with an average number of 20 million reads per sample. Sequencing resulted in 24.1 m. reads per sample on average. After quality check using FastQC [23], paired-end reads were aligned to the human reference genome (hg38) using the STAR alignment software (v 2.5.2a) [24]. After mapping, only reads that mapped to a single unique location were considered for further analysis. The mapped reads were then used to generate a count table using the feature-counts software (v 1.4.6-p5) [25] and normalized to library size [26].

### 2.8. RNA-Seq Data Analysis

The normalized RNA-Seq counts were used for differential gene expression analysis using DeSeq2 [26]. Differentially expressed genes with a false-discovery rate (FDR) < 0.01 were regarded as statistically significant. Data were further analyzed using EnrichR [27,28], GSEA [29,30] and STRING [31].

Gene set enrichment analyses were performed using EnrichR and GSEA to investigate the biological meaning of the obtained differential expressed gene lists of our RNA-Seq data. GSEA (v 4.1.0) [29,30] analysis was performed with a pre-ranked gene list according to the log2FC from differential gene expression analysis. The analysis using C2 Canonical pathways and subsets, C5 GO terms, C3 TFT, and others using gene sets from MsigDB v 7.2 [32] was done with classical weighting and 1000 permutations. Subsequent enrichment maps were produced by Cytoscape (v 3.8.0) [33] and the enrichment map app from Bader lab (v 3.3.1) [34]. The first clustering of gene sets was performed by Autoannotation from Bader lab (v 1.3.3) and later on refined by manual renaming. For some of the clusters, the manual refinement was done with the help of the GeneMania app (v 3.5.2) [35], whereas gene products coming from the differential gene expression analysis were >90% in the resulting networks.

STRING (v 11.0) [31] database was used to analyze protein associations based on curated biological pathway knowledge, scientific publications, and average GC content of the encoding transcript.

Statistical calculations were performed using R version 4.0.1 [36].

### 2.9. miRNA Transfection

The melanoma cell lines Mel Im or Mel Ju were transfected with a microRNA-146a-5p mimic or microRNA-125b mimic (both from Qiagen) using Lipofectamine RNAiMAX reagent (Life Technologies, Darmstadt, Germany) as described previously [37]. Here, 150,000 cells were seeded into the wells of a 6-well plate and incubated for 72 h. As a negative control, Allstars Negative Control siRNA (Qiagen) was used.

### 2.10. mRNA Isolation and Reverse Transcription

For analysis of basic EGR1 expression in 2D cultured NHEM and malignant melanoma cell lines (A375, Mel Wei, Mel Ju, Mel Juso, Mel Im, SkMel28, and WM3211), or EGR1 expression in 2D cultured melanoma cell lines upon treatment with miRNA mimics, total RNA was isolated using the ENZA Total RNA Kit I (Omega Bio-Tek, Norcross, GA, USA) and was transcribed into cDNA as described elsewhere [38].

### 2.11. miRNA Isolation and Reverse Transcription

For miR-146 expression analysis, microRNA was isolated from NHEMs and melanoma cell lines Mel Juso, Mel Wei, WM3211, Mel Im, and SkMel28 cultured in 2D, using the miRNeasy Mini Kit (Qiagen), and resulting purified miRNA was transcribed into micDNA as described elsewhere [39].

### 2.12. Analysis of Gene and microRNA Expression by Quantitative PCR

Quantitative reverse-transcriptase polymerase chain reaction (qRT-PCR) was performed on a Lightcycler 480 system (Roche, Rotkreuz, Switzerland). Gene expression analysis was performed using specific primer sets for the housekeeper gene β-actin and the gene of interest EGR1 (β-actin: 5′-CTACGTCGCCCTGGACTTCGAGC-3′, 5′-GATGGAGCCGCCGATCCACACGG-3′; EGR1: 5′-GGACCTGAAGGCCCTCAATAC-3′, 5′-CCTCTTGCGTTCATCGCTC-3′) as described previously [37]. For expression analysis of microRNA146-5p qRT-PCR was performed as described elsewhere [18] using the miScript II RT Kit (Qiagen) with either the corresponding miScript miRNA146-5p primer assay (Qiagen) or U6 rRNA primers as a negative control.

### 2.13. In Silico Analysis

In silico analysis of EGR1 expression level was performed using Gene Expression Omnibus (GEO) datasets (GEO profiles/GDS). The GEO dataset GDS5085/GSE42872 provides cDNA array data (Robust Multichip Average (RMA) normalized) of A375 melanoma cells harboring the BRAF V600E oncogenic mutation which were treated with the BRAF inhibitor vemurafenib (*n* = 3) or vehicle (DMSO) (*n* = 3), respectively, and was mined for EGR1 (GEO profile GDS5085/8108370) [40]. The dataset GDS3964/GSE7929 provides cDNA array data (RMA normalized) for the analysis of subcutaneous tumors or lung metastases from immunodeficient mice which were injected subcutaneously or intravenously with the poorly-metastatic A375 melanoma cell line (*n* = 11) or with highly-metastatic derivative cell lines (*n* = 21). We analyzed this dataset for EGR1 (GEO profile: GDS3964/201694_s_at, GSE7929) [41].

### 2.14. Statistical Analysis of Experimental Data

Statistical analysis was performed using the GraphPad Prism software package (GraphPad Software Inc., San Diego, CA, USA). If not further specified in the figure legends, results were expressed as the mean ± standard error of the mean (SEM). Comparisons between groups were conducted using Student’s unpaired *t*-test or one-way ANOVA, respectively. If not depicted otherwise in figure legends, the number of independent experiments was *n* ≥ 3.

## 3. Results

### 3.1. Functional Clustering of Differential Expressed Genes Reveals High Impact of Cell Culture Conditions on Gene Expression

In a recent study, we successfully used 3D alginate hydrogel culture for melanoma cells [13] and revealed that alginate was a matrix that enabled the proliferation of the melanoma cell lines while maintaining expected tumor heterogeneity.

**Figure 1:**
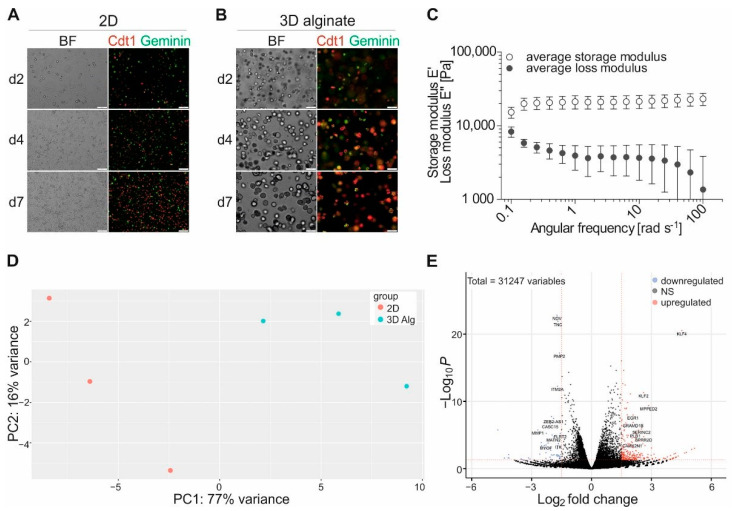
Differential phenotype and genotype of melanoma cell line Mel Im cultured in 2D versus 3D alginate. Fluorescence and bright-field images of Mel Im FUCCI after 2, 4, or 7 days of cultivation in (**A**) 2D or (**B**) 3D alginate, (Cdt1: Chromatin licensing and DNA replication factor 1), scale bars represent 100 µm; (**C**) Average storage and loss moduli ± SD determined for 0.6% alginate; (**D**) Principal Component Analysis (PCA) of RNA Seq samples; (**E**) Volcano plot of analyzed genes.

To emphasize the differences in cellular morphology and growth behavior of the cell line Mel Im in 3D alginate compared to 2D monolayer culture, we here directly compare the two conditions. Therefore, Mel Im was stably transduced with the Fluorescent Ubiquitination-based Cell Cycle Indicator (FUCCI) and cultivated either in 2D or 0.6% alginate for up to 7 days. First, bright field microscopy images were analyzed focusing on cell morphology [20]. The cells show their described spindle-like shape with only punctual cell-cell contacts in 2D culture (Figure 1A) but form dense multicellular clusters in the 0.6% alginate matrix over a cultivation period of 7 days (Figure 1B). The FUCCI reporter visualizes the current cell cycle stage by expressing the fluorescently labeled cell cycle regulators Chromatin licensing and DNA replication factor 1 (Cdt1) or Geminin in the nucleus. We quantified Cdt1-positive cells (= G1 phase) and Geminin-positive cells (= S/G2/M) as well as cells expressing both (= G1/S transition) in the respective culture condition on day 1. Cells cultured in 3D alginate showed 8.3% more cells in G1 than cells cultured in 2D and 9.9% fewer cells in the S/G2/M phase at this early time point (Figure S1). The presence of cells in G1 as well as in S/G2/M phase on the fluorescence microscopy images on days 2, 4, and 7 after seeding indicates that there is no cell cycle arrest over 7 days in 2D or 3D alginate culture, supporting the observation that cells can proliferate in both conditions. 

For tissue culture, plastics like polystyrene the Youngs modulus E defining the stiffness of a material is known to be in the GPa range at room temperature [42]. The stiffness of alginate hydrogel varies based on the dilution, crosslinking time and the ratio of guluronic acid to mannuronic acid in the alginate batch used. By dynamic mechanical analysis (DMA) we determined an average storage modulus E’ (± SD) of 0.6% (*w/v*) alginate of 21 ± 4 kPa (Figure 1C) at an angular frequency of 1 rad s^−1^. As the loss modulus E’’ was relatively low, it is to neglect for the alginate measured. The storage modulus is representative of the elastic portion of material properties in dynamic mechanical measurements. Studies have shown, that a comparison of storage modulus E’ and Young’s modulus E is valid for highly gelled samples (E’ >> E’’) [43].

To now define genes being differentially expressed in 3D versus 2D culture, we cultivated Mel Im melanoma cells for 2 days in 0.6% alginate or 2D classical cell culture as control, isolated the total RNA, and performed RNA-Seq.

After data preprocessing, mapping, and count table generation via feature Counts [25] we performed differential gene expression analysis using DeSeq2 [26] as described previously [44]. PCA (principal component analyses) notably defined the two clusters (Figure 1D) of the 3D alginate and 2D cultured samples, respectively. DESeq2 analysis (logFC > 1.5, <−1.5; adj. *p* < 0.1; differentially expressed genes (DEGs) in Table S1) resulted in 1063 upregulated and 1762 downregulated genes demonstrating significant differences in gene expression between 2D and 3D cultured cells, which are illustrated in a volcano plot depicting the log2-fold changes and statistical significance (*p*-value < 0.05, log2-fold change > 1.5 or <−1.5) of each gene (Figure 1E).

**Figure 2:**
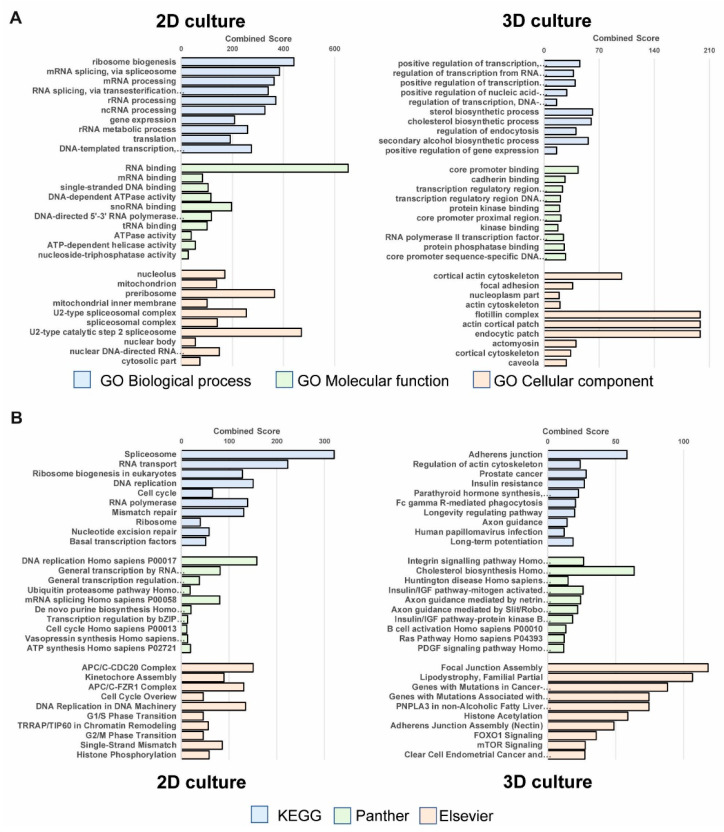
Analysis of differentially expressed genes in Mel Im in 2D culture and 3D alginate. (**A**) Top 10 results of the six enrichment analyses of GO domains and (**B**) pathway enrichment analysis. The results shown are sorted in ascending order by padj. The bars are representing the combined score.

Next, we aimed to assign biological meaning to the identified set of differentially expressed genes comparing the cultivation conditions 2D versus 3D alginate. The expression patterns were analyzed based on gene ontology (GO) and biological pathways (PW) using EnrichR (top ten results ordered by padj in Figure 2A, entire EnrichR results in Tables S2 and S3, 2825 genes with padj < 0.1; the entire list of analyzed differentially expressed genes can be found in the Table S1 (see also [45,46]). The combined score was used for the evaluation as it was previously shown to outperform other ranking metrics [27,28].

Gene ontology analysis revealed a connection between rRNA and ribosome biosynthesis, mRNA and other RNA processing and general translation (GO Biological processes), and diverse RNA binding and ssDNA-related activities (GO Molecular functions) correlating to sub-nuclear and mitochondrial compartments (GO Cellular component) in 2D cell culture. In 3D alginate cultured cells, general transcription from RNA POL II promoter in addition to some specific biosynthetic processes (GO Biological processes) are associated with transcriptional and signal transduction activities (GO Molecular functions) as well as specific processes on membrane-associated compartments (GO cellular component) (Figure 2A and Table S2). Pathway enrichment analysis results clearly show a linkage of progression through the cell cycle, DNA replication, and repair to 2D cell-cultured cells analyzing KEGG, Panther, and Elsevier pathways whereas upregulated genes in 3D alginate cultured melanoma cells significantly correlate with adherens and focal junctions, axon guidance, and other Rho GTPase associated pathways, as well as insulin/insulin-like growth factor (IGF), platelet-derived growth factor (PDGF) and epidermal growth factor receptor (EGFR) signaling (Figure 2B and Table S3). 

**Figure 3:**
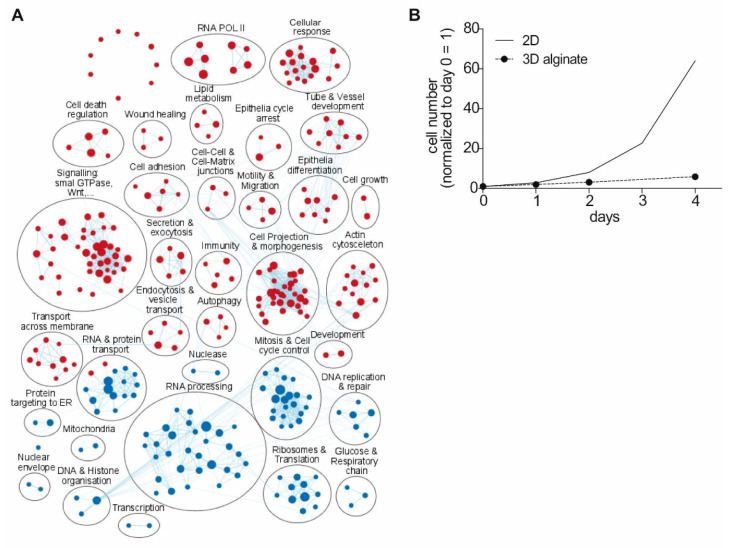
Different phenotypes in 2D and 3D alginate cultured Mel Im revealed by enrichment map analysis. (**A**) Enrichment map from GSEA of GO biological processes gene sets shows a proliferative phenotype (blue) and a principal change in cellular phenotype (red) of 2D and 3D cultured Mel Im cells, respectively. (**B**) Cell numbers of melanoma cell line Mel Im cultivated in 3D alginate over 4 days in culture (*n* = 3) were quantified from microscope images and compared to values calculated for Mel Im cultured in 2D with a doubling time of approx. 16 h. Comparison of the growth rates reveals a slower proliferation rate in 3D alginate (plotted as mean ± SD).

To visualize the functional relevance of the differentially expressed genes (DEGs), we performed classical gene set enrichment analysis (GSEA) [29,30] on the identified DEGs as a pre-ranked gene list (padj < 0.1, 2825 genes) using the calculated log2-fold changes (log2FC). Enrichment analyses were performed using MSigDB sets [47], focusing on GO biological processes (Figure 3A). Using an enrichment map as well as clustering and autoannotation [34,48], GSEA analysis revealed similar results. 2D culture samples (Figure 3A, blue nodes) show enrichment in RNA processing, stress response including DNA repair, and an increase in factors for cell cycle progression, mitosis, and cell cycle control. Furthermore, a clear increase in general factors for transcription initiation and control, translation, and nuclear transport could be found. In addition to the effects shown before on mitochondrial proteins, GSEA enrichments show an increase in respiratory chain proteins, protein assembly, and ATPase activity. In alginate 3D cultures (Figure 3A, red nodes) enrichment in GTPase signaling was found. Furthermore, gene clusters characterizing migration and locomotion, tube and epithelial organization as well as actin filaments and stress fibers could be detected. In addition to some clusters about insulin receptor (INSR), EGFR, ERBB, and fibroblast growth factor (FGF) signaling, one cluster for morphogenetic processes and one cluster for cell differentiation can be found.

Analysis of all significantly DEGs (2825 genes, padj < 0.1) in STRING validated the strong downregulation of genes involved in RNA processing (in *biological process, reactome pathways*), preribosomal and mitochondrial proteins (in *cellular component*), and cell cycle (*reactome pathways*) in 3D alginate. In addition, processes like G protein-coupled receptor signaling pathway, actin cytoskeleton organization, cell leading edge, ruffles, regulation of lipid metabolic process, cellular response to laminar fluid shear stress, and endo-/exocytosis were significantly linked to genes upregulated in 3D alginate (Table S4). Table 1 summarizes the relevant correlations of upregulated genes (top three results for enrichment score (ES), all FDR < 0.01), thereby supporting again a prominent role of small G proteins, especially of Rho GTPase.

Next, STRING [31] was used to identify publications including gene sets significantly correlating to the gene expression observed in 3D alginate (Table S5). With regards to melanoma, genes in a publication focusing on stemness of the melanoma cells significantly correlated with the transcriptomic changes observed suggesting that the detected molecular changes represent cellular plasticity [49].

Enrichment via EnrichR analysis regarding transcription factors (TF), which can be responsible for the observed gene expression signatures, resulted for example in microphthalmia-associated transcription factor (MITF), transcription factor AP-2 gamma (TFAP2C), forkhead box A2 (FoxA2), lysine demethylase 2B (KDM2B), SMAD family member 3 (SMAD3), SRY-box 9 (Sox9), SRY-box 2 (Sox2), signal transducer and activator of transcription 3 (STAT3), runt-related transcription factor 2 (RUNX2) being associated with the induction of target gene expression in 3D alginate. Lysine demethylase 5B (KDM5B), MYC proto-oncogene (Myc), MYC-associated factor X (Max), ETS proto-oncogene 1 (ETS1), E2F transcription factor 1 (E2F1), homeobox C9 (HoxC9), forkhead box P3 (FoxP3), lysine demethylase 5A (JARID1A) and POU class 3 homeobox 2 (POU3F2) were linked to targeting genes downregulated in 3D alginate compared to 2D culture (Table S6). In general, TFs associated with downregulated genes are related to cell proliferation. To analyze the functional differences in cell proliferation depending on the cultivation method, we determined the proliferation rate of Mel Im in 0.6% (*w/v*) alginate and compared it to theoretical cell numbers based on the doubling time of approx. 16h known for Mel Im in 2D monolayer culture (Figure 3B). Mel Im were cultivated in the matrix for 7 days and cells were quantified based on fluorescent image z-stacks taken on days 1, 2, and 4, respectively. For Mel Im in 3D alginate culture, we calculated a doubling time of 38 to 39 h. Due to the phenotype switching model in melanoma as one main model for heterogeneity and plasticity [50,51], we screened the EnrichR analysis for the enrichment of relevant transcription factor target genes in the CHEA database, namely genes with MITF and POU3F2 (Brn2) binding site, respectively. MITF target genes are significantly enriched in 3D cultured cells (21258399 ChIP-Seq MELANOMA Human, padj = 2.31 × 10^−25^; ChEA (experimentally validated targets) [52]), whereas POU3F2 target genes (Brn2) are significantly enriched in 2D cultured cells (20337985 ChIP-Seq 501MEL Human; padj ~ 0.025, ChEA (experimentally validated targets)). 

In summary, the analysis of cell proliferation, as well as the enrichment of relevant TFs for the phenotype switching model of 2D and 3D cultured cells, confirm a fundamentally differing phenotype.

### 3.2. Regulation of Gene Expression Level by 3D Cultivation

Changing cell culture conditions from a 2D to a 3D alginate system strongly modulates gene expression showing the high potential of plasticity of melanoma cells to adjust to changes in the microenvironment. As this feature is also observed in vivo and is of strong importance for melanoma progression as well as therapy resistance, we aimed at understanding the changes in more detail and to define their relevance for the in vivo situation. 

Next, we focused on individual genes most significantly differentially expressed in 0.6% (*w/v*) alginate compared to 2D cell growth (padj < 0.01, log2FC > 2 or <−1.5; Table 2). 

Here, 12 genes, including early growth response (EGR1), Kruppel-like factor 4 (KLF4), metallophosphoesterase domain containing 2 (MPPED2), Kruppel-like factor 2 (KLF2), phospholipase B1 (PLB1), GRAM domain containing 1B (GRAMD1B) and calcium/calmodulin-dependent protein kinase II inhibitor 1 (CAMK2N1) were found to be induced in 3D cultured Mel Im cells, whereas 13 genes, including cellular communication network factor 3 (NOV), tenascin C (TNC), cadherin 2 (CDH2), matrix metallopeptidase 1 (MMP1), protocadherin 7 (PCDH7) and peripheral myelin protein 2 (PMP2), were found to be reduced in their expression.

As KLF2 is a transcription factor known to mediate the tumor suppressor gene p53, we also looked at CDKN1A, a known direct target of p53, and found it to be significantly induced in 3D alginate (logFC 0.567, padj = 0.043).

**Figure 4:**
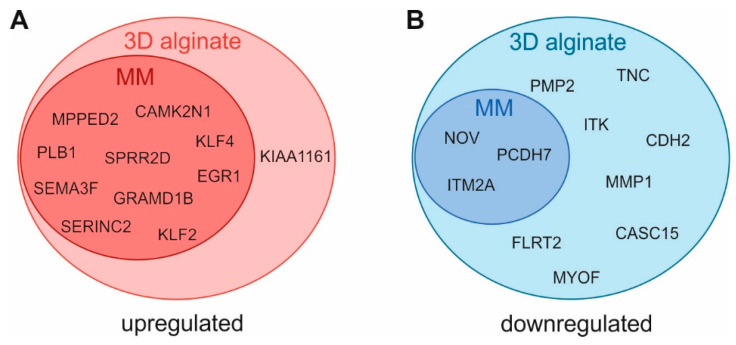
Overlap of significantly deregulated genes in 3D alginate vs. 2D with differentially expressed genes in melanoma (MM) vs. melanocytes (NHEM). (**A**) Genes upregulated in MM and/or 3D alginate. (**B**) Genes downregulated in MM and/or 3D alginate.

Interestingly, the most strongly differentially expressed genes are known to play a crucial role in cancer and seem to be linked to cellular plasticity. Therefore, we compared the gene expression patterns observed in 3D alginate with cDNA array data published by Dietrich et al., where gene expression patterns of melanocytes (NHEM) and melanoma (MM) were analyzed [53] (3 genes are not resembled in the array). With 10 out of 11 most significantly induced genes in 3D alginate being also significantly induced in melanoma cells compared to NHEM, we revealed a strong correlation of genes upregulated in 3D alginate with genes induced during melanoma development (Figure 4A).

In contrast, we observed that only 3 out of 11 genes downregulated in 3D cultivation also showed downregulation during melanoma development (Figure 4B). NOV, PCDH7, and integral membrane protein 2A (ITM2A) showed a strong reduction also comparing NHEM to MM. All others demonstrate an increased expression in melanoma development, some even strongly like PMP2 (5.7-fold), TNC (10.2-fold), MMP1 (65.6-fold), and CDH2 (10.2-fold).

### 3.3. Role of EGR1 in Melanoma 

As mentioned above, one of the most significantly induced genes in 3D alginate and MM was EGR1. As several studies already suggested EGR1 having a role in neuronal plasticity, this gene was of strong interest for our further analysis of melanoma cell plasticity/heterogeneity.

**Figure 5:**
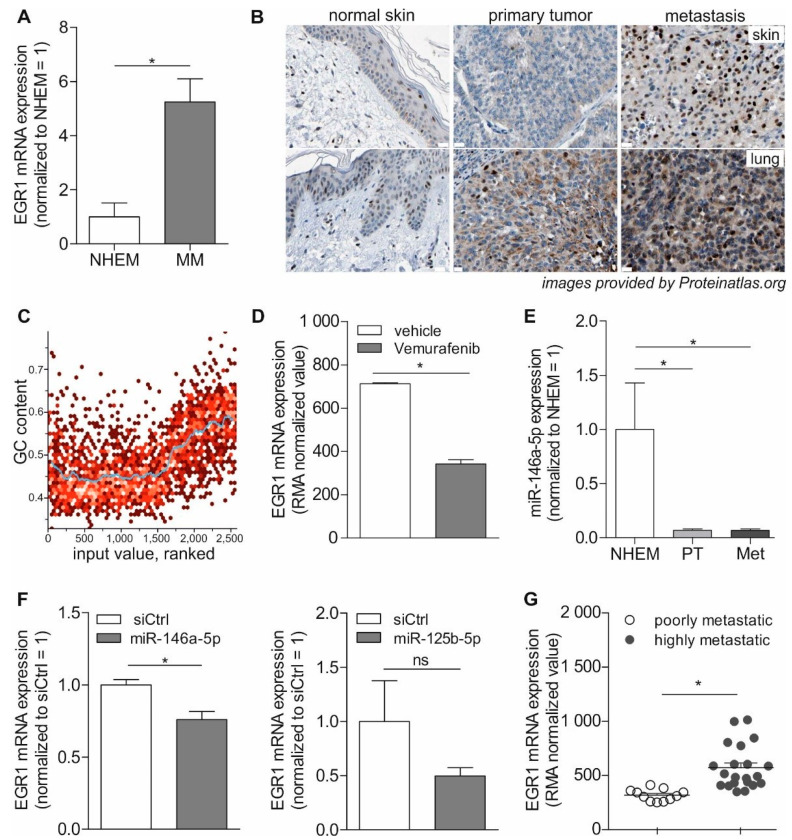
EGR1 expression and regulation in melanoma in 2D culture or tissue sections. (**A**) qRT-PCR analysis shows significantly increased EGR1 mRNA expression in seven malignant melanoma cell lines (MM; *n* = 14) compared to primary melanocytes (NHEM; *n* = 3). (**B**) EGR1 immunohistological staining of skin or melanoma tissue (publicly available data from Proteinatlas.org [54,55]) shows increasing staining intensity and nuclear staining from normal melanocytes in the basal layer of the epidermis (Patient-ID: 2156; 3403) over primary tumor (PT; Patient-ID: 3611; 2900) to metastasis (Met, Patient-ID: 1020 (skin); 2317 (lung)) [56,57]. (**C**) STRING analysis of the ranked values of differential gene expression (2D vs. 3D alginate) revealed a correlation with GC-content of associated genes (Pearson’s r-value: 0.547, Pearson’s *p*-value: 10^−32^, BP-R^2^: 0.399 (very high)). (**D**) Affymetrix array data showing a decrease in EGR1 mRNA expression in the melanoma cell line A375 treated with the BRAF inhibitor vemurafenib (*n* = 3) compared to A375 treated with vehicle (0.1% DMSO; *n* = 3) (Gene Expression Omnibus (GEO) Profiles: GDS5085/8108370, GSE42872, [40,58]) (RMA: Robust Multichip Average). (**E**) qRT-PCR analysis of EGR1 regulating miR146a expression is significantly decreased in melanoma cell lines from the primary tumor (PT) or metastasis (Met) compared to NHEM. (**F**) qRT-PCR analysis of melanoma cell line Mel Im upon treatment with miR146a mimic (*n* = 3), and Mel Ju or Mel Im upon treatment with miR-125b mimic (*n* = 2) reveal downregulation of EGR1 mRNA. (**G**) Affymetrix array data showing EGR1 mRNA expression increases significantly from poorly metastatic melanoma cell line A375 (*n* = 11) to highly metastatic derivatives (*n* = 21) (GEO Profiles: GDS3964/201694_s_at, GSE7929, [41,59]). * *p* < 0.05.

We confirmed EGR1 induction in MM compared to primary melanocytes (NHEM) by qRT-PCR analysis of the EGR1 mRNA expression in seven different melanoma cell lines (A375, Mel Wei, Mel Ju, Mel Juso, Mel Im, SkMel28, and WM3211) and NHEM cultured in 2D (Figure 5A).

Next, the EGR1 protein expression was examined, using publicly available data (Proteinatlas.org [54]), on EGR1 immunohistological stainings of healthy skin, melanoma primary tumor, or melanoma metastasis tissue, and different staining patterns were observed (Figure 5B). In healthy skin, no EGR1 expression was detected in the melanocytes (lying in the basal layer of the epidermis), whereas other cell types like fibroblasts (in the dermis) are positive. In primary tumor tissues, weak and mostly cytoplasmic staining for EGR1 was observed in the tumor cells. In the metastases, staining shifted to a more intense and nuclear staining. In summary, increasing EGR1 mRNA and protein expression during melanoma progression are a common feature in melanoma cells.

Further information on EGR1 was gained by exploring the gene set enrichment analysis output data of EnrichR. Enrichment analysis of upregulated genes regarding the CORUM database revealed *EGR-EP300 complex (human)* (*p* = 0.0028, combined score: infinity, Table S6) to be linked to cultivation in 3D alginate. Furthermore, we annotated known EGR1 target genes inside the enrichment map of the GSEA result and revealed a clear connection towards enriched gene sets of the 3D alginate cultured cells (Figure S2).

EGR1 is known to bind to GC-rich DNA motifs via its C2H2-type zinc fingers. Interestingly, in STRING analysis the ranked values of differential gene expression of 2D or 3D alginate cultured cells revealed a significant correlation to the GC content at the loci of the associated proteins (Pearson’s r value: 0.547, Pearson’s *p*-value: 10^−32^, BP-R^2^: 0.399 (very high)) (Figure 5C). 

EGR1 has previously been shown to react to mechanical stimuli. As studies in melanoma cells could demonstrate that Rho activators (calpeptin and ilomastat) regulating actin dynamics activate EGR1 expression (Gene Expression Omnibus (GEO) Profiles: GDS5670/8108370, GSE52246 [60]), we analyzed these aspects in our setting. RHOB expression was determined to be induced (log2FC 1.57; padj = 2.508 × 10^−10^) in 3D alginate and cytoskeletal regulation by Rho GTPase were pathways linked to deregulated genes (Table 2, Table S4).

Additionally, EGR1 is known to be induced by RAF/Ras signaling in melanoma. To confirm this, we analyzed a publicly available Affymetrix array data set by Parmenter et al. [57] for EGR1. Here, A375 melanoma cells harboring the BRAF V600E oncogenic mutation, and therefore having constitutively active Ras/RAF signaling, were treated with the highly specific BRAF mutant inhibitor vemurafenib or control vehicle, respectively, and displayed strong negative effects of BRAF inhibitors on EGR1 expression (GEO Profiles: GDS5085/8108370, GSE42872 [40], Figure 5D). Furthermore, own cDNA array data confirm 3.7-fold EGR1 induction in melanocytes after lentiviral transduction with mutated BRAF (data in [19]).

In addition to RAS/Raf signaling, interestingly also miRNAs are known to regulate EGR1 expression. As miR146a was previously shown to regulate EGR1 [61], we analyzed the miR146a expression in NHEM and five melanoma cell lines from the primary tumor (Mel Juso, Mel Wei, WM3211) and metastasis (Mel Im, SkMel28) by qRT-PCR and confirmed its downregulation in melanoma compared to melanocytes (Figure 5E) [18,62]. Further miR-125b, which we revealed previously to be especially reduced in metastasis and progression [62,63], was shown to regulate EGR1 [64,65]. Transfection of miR mimics of miR146a or miR125b in melanoma cell lines Mel Im or Mel Ju resulted in downregulation of EGR1, however, for miR1215b this regulation was not significant (Figure 5F).

Wu et al. [66] revealed regulation of Insulin-Like Growth Factor-1 Receptor (IGF1R) expression by EGR1. IGF1R is also found to be induced in 3D alginate in our data (logFC 0.48; padj = 0.0042; Supplementary Table S1), further, ARCHS4 and KEA analysis for pathway regulation revealed several genes known to be regulated by IGF1R in the differentially induced genes (IGF1R human kinase ARCHS4 co-expression, padj = 6.89 × 10^−6^; PANTHER Insulin/IGF pathway-protein kinase B signaling cascade Homo sapiens P00033 (*p* = 0.08)).

Further, Wu et al. [67] showed that ZEB1 is a direct target of EGR1 together with Snail. We found the ZEB1 expression to be induced in 3D alginate (logFC 0.98, *p* = 0.0014).

Next, we wanted to evaluate the impact of EGR1 expression on metastasis in vivo. In a study by Xu et al. [59] a xenograph melanoma model was used to define gene expression changes correlating with the aggressiveness of human melanoma metastases. We analyzed the publicly available Affymetrix array dataset with regards to EGR1 and revealed a significant correlation of EGR1 expression with the aggressiveness of melanoma metastases (Figure 5G; GEO Profiles: GDS3964/201694_s_at, GSE8401 [41], *p* = 4.48 × 10^−4^ log2FC 0.7632849).

## 4. Discussion

Three-dimensional cell culture methods are on the rise as they hold the potential to better mimic the physiological microenvironment of cells compared to 2D tissue culture plastics. Matrix stiffness, cell types, and arrangements can be designed as desired and thereby surpass 2D cultures concerning hierarchy and complexity. However, to be able to fully understand and control such 3D models, it is indispensable to unravel genes regulated as well as their impact on cell behavior in varying culture settings. Despite the huge interest in these novel model systems, broad gene expression analyses of cells from 3D hydrogel cultures are rare.

For the first time, we performed RNA-Seq of melanoma cells cultured in 0.6% (*w/v*) alginate compared to common 2D monolayer culture with subsequent unbiased bioinformatics analysis and were able to detect 2825 deregulated genes in total. Alginate takes the 3D environment into account and allows for more in vivo-like growth of cells in clusters with actual cell-cell contacts. Thereby, it resembles the tumor situation more precisely than a flat tissue culture plastic dish. Various factors influence tumor growth in vivo. However, careful estimation of a doubling time for Mel Im in mice in vivo based on tumor sizes measured by Dietrich et al. [38] shows that tumor size doubling takes approx. 2–3 days (analyzing day 28–35, the main growth period) up to around one week (after the tumor reached a certain size). This hints towards slower cell doubling in vivo compared to in vitro melanoma culture, being consistent with the decreased cell proliferation rate we observed in 3D alginate cultured cells.

Functional clustering of the deregulated genes revealed the crucial impact of cell culture conditions on gene expression. As described above in detail, Gene Ontology, pathway, STRING, and GSEA analyses revealed a significant correlation of 2D cultured cells with e.g., cell cycle progression, DNA replication, and repair, whereas cells from alginate culture are associated with e.g., adherens and focal junctions, migration, and locomotion as well as various Rho GTPase associated pathways. In general, transcription factors associated with downregulated genes in 3D alginate were found to be related to cell proliferation, agreeing with the decreased proliferation rate determined for Mel Im in 3D alginate. Until today only two studies, both by Darnell et al., using RNA-Seq as unbiased transcriptome analysis to define cells in 3D alginate hydrogel were performed [68,69]. Darnell et al. used RNA-Seq to analyze the transcriptome of mesenchymal stem cells (MSC) in alginate of different stiffness, where the "intermediate" initial elastic modulus of 18 kPa is in the same range as the stiffness of 21 ± 4 kPa we measured for the 0.6% alginate hydrogel we used in our study. As no analysis of cells on 2D tissue culture plastic was performed by Darnell et al. and due to the different cell types used, a direct comparison of our data is not possible. However, several genes they observed to be regulated by stiffness like the transcription factor hubs of proliferation modulating transcription factors (Myc, E2Fs, Jun) and e.g., Nav2 are supported by our analysis.

We observed gene clusters for morphogenetic processes and cell differentiation in the GSEA analysis, clearly visualizing the strong phenotype change of the cells taking place in the 3D alginate after 2 days of culture

This phenotype change is reflected by the highly differential gene expression patterns of the same melanoma cell line in the two different culture conditions, and impressively shows the great potential of melanoma cells for tumor plasticity, to adjust to changes in their microenvironment. Our STRING analysis revealed that publications including gene sets significantly correlating to the gene expression observed in 3D alginate focused on cellular plasticity, stemness, pluripotency, and metastasis, indicating the relevance of the deregulated genes in these processes.

Interestingly, this connection could be further underlined by analyzing the most significantly deregulated genes in 3D alginate, which are known to play crucial roles in cancer and cellular plasticity in general. Moreover, we were able to show a significant correlation of genes most strongly induced in 3D alginate, with genes induced in malignant melanoma compared to primary melanocytes, including KLF2, KLF4, and EGR1. KLF2 for example is a transcription factor known to control the G1/S transition of the cell cycle by mediating the tumor suppressor gene p53 [70]. Interestingly, in our study also CDKN1A (p21), a known direct target of p53, is significantly induced in 3D. KLF4 is known to regulate key transcription factors during embryonic development and to play an important role in maintaining embryonic stem cells and preventing differentiation. EGR1 (alias KROX24, NGFIA or ZIF268), induced in 3D alginate, is an immediate early expressed gene containing a DNA-binding domain functioning as a transcriptional regulator [71]. It is important for numerous physiological processes including synaptic plasticity, wound repair, inflammation, and differentiation.

The overlap of downregulated genes in 3D alginate with genes downregulated in melanoma compared to melanocytes was not as matching as expected. However, the gene set is also composed of highly relevant players in cancer and cellular plasticity. The downregulated genes TNC and MMP1 are known to be commonly induced in cancer, the same is true for PMP2 and CDH2 [72,73]. These findings indicate a regulation of genes involved in the development/progression/plasticity of melanoma cells depending on the specific microenvironment. Potentially, these genes are modulated in defined environments mimicking different gene expression patterns of cell stages on the way from the primary tumor site to newly formed metastasis.

Still, the role of the genes most significantly deregulated in 3D alginate in the development, progression, and plasticity of melanoma remains to be examined. As mentioned above, EGR1 has already been suggested to play a role in neuronal plasticity. Due to the same origin of melanocytes and nerve cells, we focused on the role of EGR1 in melanoma cell plasticity and heterogeneity and indeed we found EGR1 to be increased in melanoma cell lines and tissue compared to healthy melanocytes. EGR1 stained tissue sections supported the relevance of this transcriptional regulator in vivo and suggested that EGR1 protein can be found in most melanoma cells, in addition, activation and nuclear translocation is of importance for metastasis.

The importance of EGR1 was further supported by revealing the association of upregulated genes with the EGR-EP300 protein complex by CORUM database analysis and with EGR1 target genes in general by GSEA analysis. Consistent with these observations, STRING analysis revealed a significant correlation of the ranked values of differential gene expression to the GC content at the loci of the associated proteins, suggesting that GC-regulated expression was positively influenced by 3D-culturing resulting in induced expression of the corresponding genes. This is in agreement with our finding that EGR1 was strongly induced by 3D alginate culture. EGR1 binds to DNA motifs 5′-GCG(T/G)GGGCG-3′ via its C2H2-type zinc fingers independent of the cytosine methylation status [74,75]. Potentially, strong induction of EGR1 as observed in our 3D cultivation also supports the downregulation of proliferation in alginate as Huang et al. [76] observed effects of EGR1 on cellular proliferation.

EGR1 activity can be regulated by several factors in our 3D alginate setting, including mechanosensation, RAF/Ras signaling, and miRNAs. EGR1 is known to react to mechanical stimuli in several settings, e.g., mechanical stretch stimulated EGR1 expression on mRNA and protein level in vascular smooth muscle cells [66,77,78]. In our analysis of deregulated pathways also cell membrane, ruffing, cellular response to laminar fluid shear stress were observed to correlate with transcriptomic changes further supporting to concentrate on EGR1 effects in melanoma. Further, a study by Kunnen et al. [79] documented that fluid shear stress regulates EGR1. In addition to EGR1, also KLF4 and KLF2, both significantly induced in 3D alginate in our study, are known to be mechanosensitive transcription factors. These data demonstrate that the setting in 3D alginate led to mechanosensing and cytoskeletal reactions in melanoma cells. As adhesion of the cells to alginate is not possible based on missing cell adhesion molecules binding alginate, induction of mechanical stretch increasing EGR1 transcription is rather unlikely. Other studies revealed that also dynamic compression can activate EGR1 transcription in 3D cultures of mouse primary chondrocytes [80], these effects could be responsible for EGR1 induction in 3D alginate melanoma cultures. As addressed before, Rho activators regulating actin dynamics activate EGR1 expression [60]. This is consistent with the finding that RHOB expression was induced in 3D alginate, with our enrichment analyses demonstrating a prominent role of small G proteins, especially of Rho GTPase, and with the fact that cytoskeletal regulation by Rho GTPase were pathways linked to deregulated genes. This is also in agreement with the known induction of EGR1 via RAF/Ras signaling in melanoma, which we could also verify by analyzing a publicly available dataset and datasets previously published by our group [19,58]. In 3D alginate, stimulation of the RAS/RAF pathway could be based e.g., on EGF, which expression we found to be induced in 0.6% (*w/v*) alginate (logFC 1.03; padj = 1.78 × 10^−5^). Tarcic et al. [81] revealed that the earliest TF induced by EGF was EGR1. This is supported by the finding that both, EGR1 and KLF4, were shown to be upregulated via ERK activation [82] and are known to be STAT3 targets [83]. 

We aimed to assign a functional role to EGR1 in melanoma. Our data linking EGR1 with cancer progression is supported by other studies showing that EGR1 overexpression in some cancer types directly promotes cancer progression and tumor growth by increasing the expression and secretion of growth factors, cytokines, and matrix metalloproteases, initiating the modification of extracellular matrix proteins [84,85]. Interestingly, findings suggest that KLF2 and EGR1 cooperate for the activation of the EMT program in response to mechanosensation [86].

The observed induction of ZEB1 in 3D alginate is in agreement with the study by Wu et al. [67], showing that ZEB1 is a direct target of EGR1 together with Snail. Tarcic et al. [81] demonstrated that induction of EGR1 induces a transcription program leading to cell migration and Tang et al. [87] supported a role for EGR1 in breast cancer metastasis.

In melanoma, a link between stemness and migratory potential was suggested (reviewed in [88]). Based on the molecular signatures observed in our study, this link could be relevant for EGR1 in melanoma, being further induced by 3D culturing in 0.6% (*w/v*) alginate and therefore enhancing the number of quiescent, stem cell-like cells. In glioma, EGR1 has been linked to the self-renewal of brain tumor-initiating cells [89,90,91], indicating an implication in the maintenance of niche populations. Due to the same embryonic origin of the neural crest, this role could be also assumed for melanoma supported by the data of this study. In addition, EGR1 was shown to play a role in hematopoietic stem cell quiescence critical for the maintenance of these stem cells [92]. Remarkably, also CDKN1A (p21) is relevant in this process [93], which we could show to be significantly induced in melanoma 3D alginate culture. Interestingly, also Ma et al. [94] for liver cancer and Katakam et al. [95] for colon cancer observed a role of EGR1 in stabilizing stem cells properties and metastasis and tumorigenic potential.

Compared to patient-specific tumor organoid models which are gaining more and more attention in the field of 3D cancer models (reviewed in [96]), the 3D cultivation of tumor cell lines in hydrogels like the alginate used in this study is easier with regards to handling and model complexity. In general, the great advantage of 3D hydrogel cultures is the possibility to mimic different microenvironments in terms of stiffness, matrix composition, and stromal cells. This enables one to study the direct influence of different microenvironmental factors on the behavior of different cell types, e.g., in different stages of metastasis, while organoids have strict requirements for their matrix, are more heterogeneous, and hence, difficult to control in the microenvironment. In this study, we did not focus on the generation of a complex model in the first place but wanted to examine the impact of 3D cell cultivation in alginate on melanoma cells in an isolated manner. In further studies, it will be of great interest to change the matrix or include further cell types and unravel molecular changes happening caused by these microenvironmental changes. Combining 3D cell cultivation in hydrogels with automated and controlled deposition, e.g., by bioprinting, will enable the design of highly complex structures and models, mimicking the in vivo situation in the patient even better.

However, with our study, we deliver a basic, yet fundamental RNA-Seq data set revealing a broad range of changes induced in melanoma by implementing a culture switch from 2D to 3D alginate and highlight the possibility to detect novel target genes by combining 3D cell culture and unbiased whole transcriptome analysis.

## 5. Conclusions

In summary, using 3D cell culture models, like the one described in our study, help to influence the plasticity and heterogeneity of tumor cells, here in melanoma. We revealed an impact on cell cycle, RAS signaling, GTPase signaling, cell spreading, membrane ruffles, actin cytoskeleton formation, endocytosis, and phospholipid metabolism by controlled modulation of the tumor cell microenvironment revealing the marked impact of the extracellular compartment on the plasticity of the tumor cells. By in-depth analysis of the changes in the transcriptome, EGR1 was identified to play a role in melanoma plasticity.

The possibility to modulate and define the microenvironment will lead to further specific models of e.g., certain sites of distant metastasis, certain organ-specific conditions, defined adherence of the tumor cells, etc. As shown in this study, inducing and analyzing the heterogeneity of tumor cells in these highly defined settings like 3D alginate helps to unravel important regulators of melanoma development and progression. Correlation of the cellular phenotype in 3D with gene expression study in tumors samples, especially with upcoming single-cell RNA-Seq, will help to adapt the 3D models to certain in vivo settings in the future making these models also highly attractive for therapeutic studies with potentially higher relevance than 2D culturing with regards to the in vivo situation.

## Figures and Tables

**Table 1:** Top three results based on the enrichment scores (ES) by STRING analysis. STRING analysis using GO terms, KEGG, and Reactome pathways as well as keywords resulted in similar enrichment terms for 3D alginate culture as the previous analysis (see also Table S4). It can be emphasized that especially terms regarding morphology and functions of plasma membranes in combination with small G-protein activity were enriched, in particular for Rho GTPase.

	Term Description	ES		Term Description	ES
GO Biological Process	negative regulation of heterotypic cell-cell adhesion	4.7	Reactome	Rho GTPase cycle	1.2
	cellular response to laminar fluid shear stress	4.7		signaling by GPCR	0.9
	positive regulation of nitric oxide biosynthetic process	3.9		GPCR downstream signaling	0.9
GO Molecular Function	Ras guanyl-nucleotide exchange factor activity	1.2	KEGG Pathways	cAMP signaling pathway	1.4
	RNA polymerase II regulatory region sequence-specific DNA binding	1.0		axon guidance	1.3
	RNA polymerase II regulatory region DNA binding	1.0		regulation of actin cytoskeleton	1.0
GO Cellular Component	leading-edge membrane	1.1	Keywords	guanine-nucleotide releasing factor	1.2
	site of polarized growth	1.1		SH3 domain	1.1
	ruffle	1.0		actin-binding	0.9

**Table 2:** Most significantly differentially regulated genes in Mel Im cells cultured in 3D alginate (“upregulated”) compared to 2D (“downregulated”).

	Gene Symbol	Official Full Name	Log2FC		Gene Symbol	Official Full Name	Log2FC
upregulated	KLF4	Kruppel-like factor 4	4.5	downregulated	MMP1	matrix metallopeptidase 1	−2.7
	MPPED2	metallophosphoesterase domain containing 2	2.9		MYOF	myoferlin	−2.3
	SPRR2D	small proline-rich protein 2D	2.6		CASC15	cancer susceptibility 15	−2.1
	KLF2	Kruppel-like factor 2	2.6		PCDH7	protocadherin 7	−2.0
	SERINC2	serine incorporator 2	2.5		CDH2	cadherin 2	−2.0
	KIAA1161	myogenesis regulation glycosidase	2.2		MATN2	matrilin 2	−1.9
	SEMA3F	semaphorin 3F	2.2		ZEB2-AS1	ZEB2 antisense RNA 1	−1.9
	PLB1	phospholipase B1	2.2		NOV/CCN3	cellular communication network factor 3	−1.7
	GRAMD1B	GRAM domain containing 1B	2.1		ITM2A	integral membrane protein 2A	−1.7
	EGR1	early growth response 1	2.1		TNC	tenascin C	−1.7
	CAMK2N1	calcium/calmodulin-dependent protein kinase II inhibitor 1	2.0		ITK	IL2 inducible T cell kinase	−1.7
	NANOGP4	Nanog homeobox pseudogene 4	2.0		PMP2	peripheral myelin protein 2	−1.6
					FLRT2	fibronectin leucine-rich transmembrane protein 2	−1.6

## Data Availability

The data presented in this study are openly available in GEO Profiles (GDS3964/201694_s_at, GSE7929, GDS5085/8108370, GSE42872; [40,41,58,59]) and Supplementary Table S1.

## Supplementary Materials

The following are available online at https://www.mdpi.com/article/10.3390/cancers13164111/s1, Figure S1: Quantification_FUCCI_d1, Figure S2: EnrichmentMap_EGR1Annotation, Table S1: DiffReg_all, Table S2: GOenrich, Table S3: PWenrich, Table S4: String_GO_PW, Table S5: String_PMID, Table S6: TFenrich.
