# Supplementary material for: Molecular Changes Induced in Melanoma by Cell Culturing in 3D Alginate Hydrogels
Source: Cancers (Basel). 2021 Aug 15;13(16):4111. doi: 10.3390/cancers13164111 (PMC8394053; doi:10.3390/cancers13164111)
Supplement: Supplementary file 1 [file cancers-13-04111-s001.zip › cancers-1289663-supplementary-figures.pdf]

Supplementary Materials: Molecular Changes Induced in Melanoma by Cell Culturing in 3D Alginate Hydrogels

Melanie Kappelmann-Fenzl, Sonja K. Schmidt, Stefan Fischer, Rafael Schmid, Lisa Lämmerhirt, Lena Fischer, Stefan Schrüfer, Ingo Thievessen, Dirk W. Schubert, Alexander Matthies, Rainer Detsch, Aldo R. Boccaccini, Andreas Arkudas, Annika Kengelbach-Weigand and Anja K. Bosserhoff

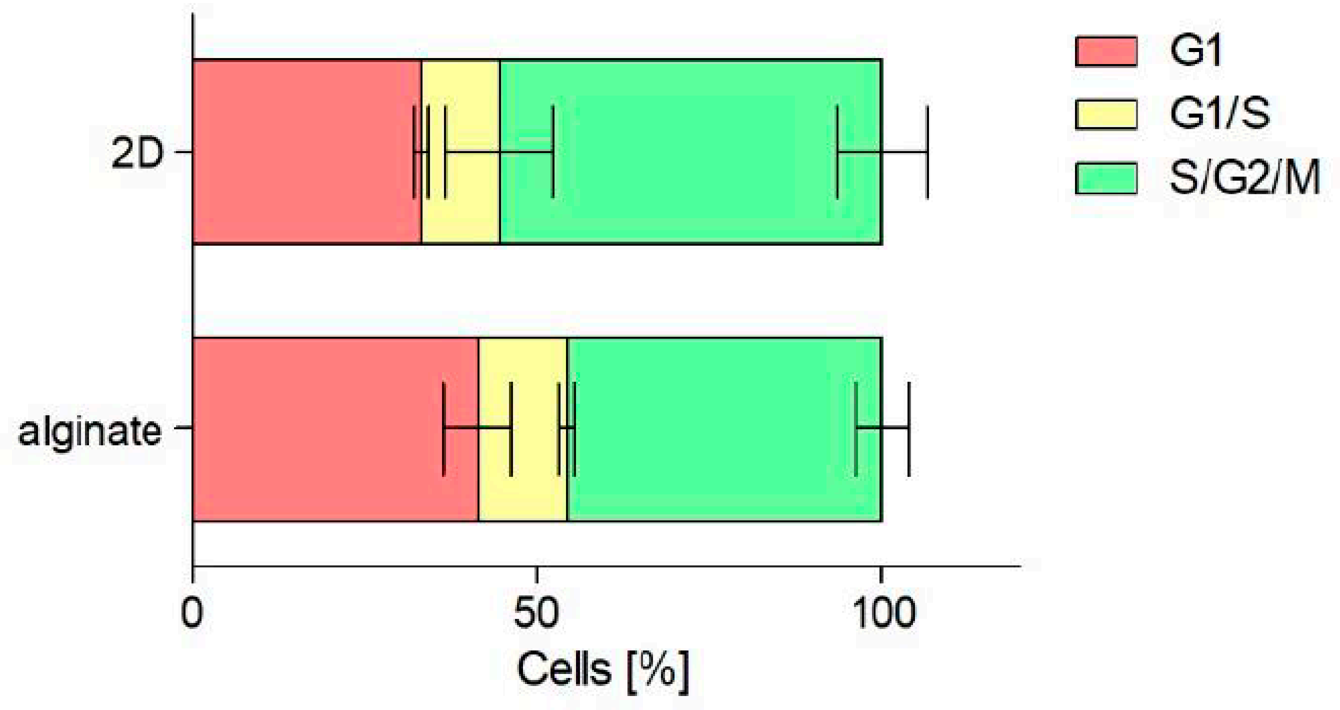

Figure S1. Quantification of cell cycle state of Mel Im FUCCI cells, determined from microscopy images taken on day 1 after seeding in 2D or 3D alginate, respectively.

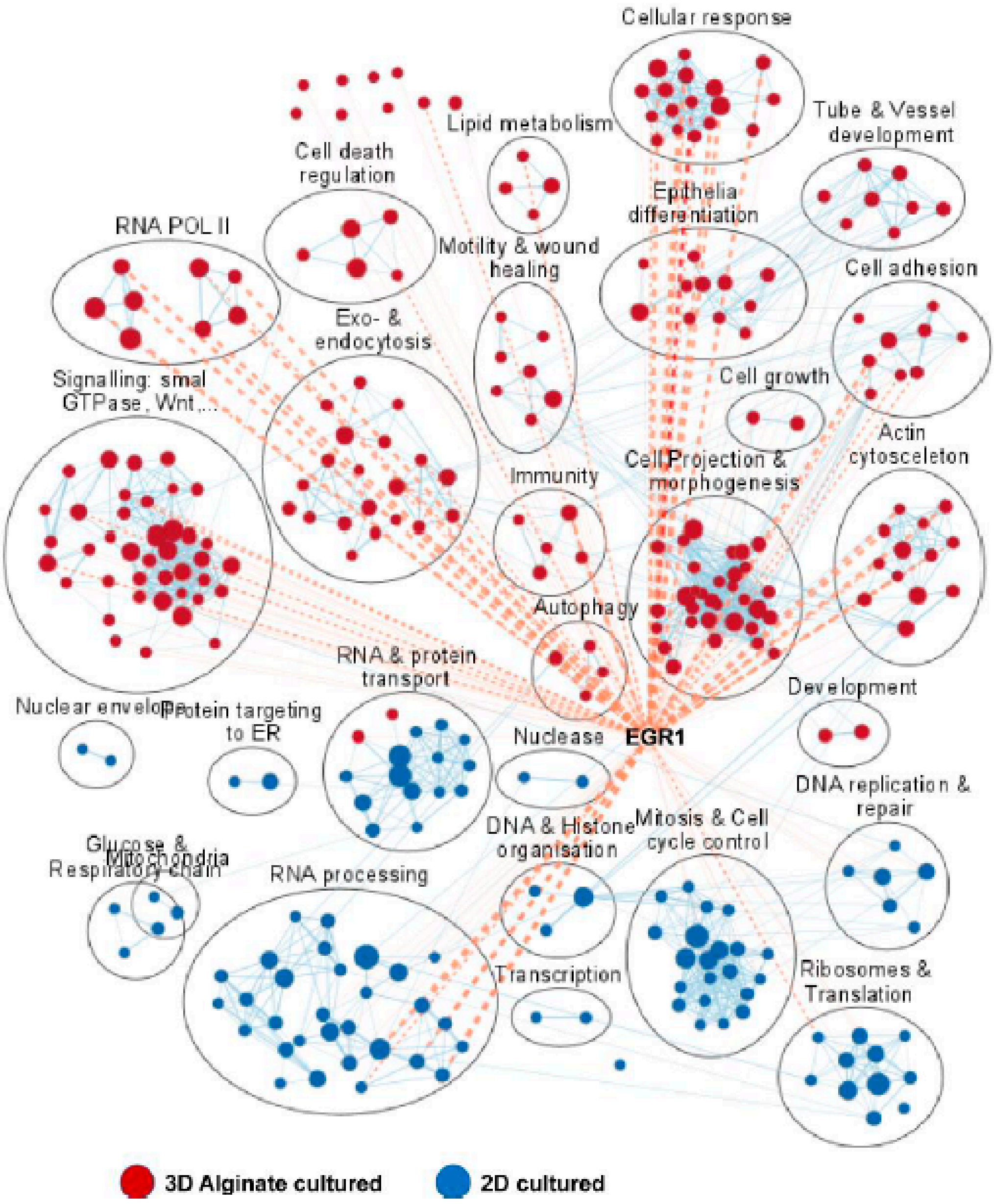

Figure S2. Enrichment map from Figure 2C with annotation of known EGR1 target genes.
